# Correction: Can computer simulation support strategic service planning? Modelling a large integrated mental health system on recovery from COVID-19

**DOI:** 10.1186/s13033-024-00657-3

**Published:** 2024-12-31

**Authors:** Livia Pierotti, Jennifer Cooper, Charlotte James, Kenah Cassels, Emma Gara, Rachel Denholm, Richard Wood

**Affiliations:** 1 NIHR Bristol Biomedical Research Centre, University of Bristol, Bristol, UK; 2 NIHR Health Protection Research Unit in Behavioural Science and Evaluation, University of Bristol, Bristol, UK; 3 Bristol, North Somerset and South Gloucestershire Integrated Care Board, UK National Health Service, Bristol, UK; 4 HDR UK Southwest, Bristol, UK

**Correction: International Journal of Mental Health Systems (2024) 18:12**

10.1186/s13033-024-00623-z

Following publication of the original article, the authors would like to correct the funding section.

The sentence currently reads:

This work was partially supported by Elizabeth Blackwell Institute.

The sentence should read:

This work was partially supported by Elizabeth Blackwell Institute. Wellcome funder grant number 204,813/Z/16/Z.

The original article has been corrected.

