# Supplementary material for: Can computer simulation support strategic service planning? Modelling a large integrated mental health system on recovery from COVID-19
Source: Int J Ment Health Syst. 2024 Mar 7;18:12. doi: 10.1186/s13033-024-00623-z (PMC10918932; doi:10.1186/s13033-024-00623-z)
Supplement: Supplementary file 1 — Additional file 1: Table S1. Services type for primary and secondary care mental health outpatients services. Figure S1. Summary of Weekly Queue size for services modelled post ‘lockdown’ (mean and 95% confidence bands) for the simulation scenarios A. Figure S2. Summary of Weekly Queue size for services modelled post ‘lockdown’ (mean and 95% confidence bands) for the simulation scenarios B. [file 13033_2024_623_MOESM1_ESM.docx]

**Additional tables and figures**

Table S1: Services type for primary and secondary care mental health outpatients services.

| **Service Type Referred To** | **mental health group** |  | **Service Type Referred To** | **mental health group** |
| --- | --- | --- | --- | --- |
| Prescriptions for anxiety and/or depression | L2 High intensity |  | Psychiatric Liaison Service | L4 MHP General |
| General practice | L2 Low intensity |  | Psychotherapy Service | L4 MHP General |
| nurse | L2 Low intensity |  | Psychological Therapy Service (non IAPT) | L4 MHP General |
| unknown | L2 Low intensity |  | Early Intervention Team for Psychosis | L4 MHP General |
| Health care assistant | L2 Low intensity |  | Young Onset Dementia Team | L4 MHP General |
| other | L2 Low intensity |  | Asylum Service | L4 MHP General |
| Mental Health pharmacist / paramedic / social prescriber | L2 Low intensity |  | Individual Placement and Support Service | L4 MHP General |
| Advanced nursing practice | L2 Low intensity |  | Problem Gambling Service | L4 MHP General |
| Assessment | L3 IAPT Assessment |  | Rough Sleeping Service | L4 MHP General |
| Review | L3 IAPT Assessment |  | Community Eating Disorder Service | L4 MHP Specialist |
| Follow-up appointment | L3 IAPT Assessment |  | Substance Misuse Team | L4 MHP Specialist |
| Improving Access to Psychological Therapies low intensity therapy (regime/therapy). | L3 Social prescribing |  | Acquired Brain Injury Service | L4 MHP Specialist |
| Applied relaxation (regime/therapy) | L3 Social prescribing |  | Criminal Justice Liaison and Diversion Service | L4 MHP Specialist |
| Guided self-help using book (regime/therapy) | L3 Social prescribing |  | Prison Psychiatric In reach Service | L4 MHP Specialist |
| Other Mindfulness-based therapy (regime/therapy) | L3 Social prescribing |  | Personality Disorder Service | L4 MHP Specialist |
| Non-guided self-help using book (regime/therapy) | L3 Social prescribing |  | Community Team for Learning Disabilities | L4 MHP Specialist |
| Guided self-help using computer (regime/therapy) | L3 Social prescribing |  | Epilepsy/Neurological Service | L4 MHP Specialist |
| Treatment Applied relaxation (regime/therapy) | L3 Social prescribing |  | Specialist Parenting Service | L4 MHP Specialist |
| IAPT Treatment | L3 IAPT Therapy |  | Forensic Mental Health Service | L4 MHP Specialist |
| Treatment Psychoeducation | L3 IAPT Therapy |  | Forensic Learning Disability Service | L4 MHP Specialist |
| Counselling for depression (procedure) | L3 IAPT Therapy |  | Autistic Spectrum Disorder Service | L4 MHP Specialist |
| Cognitive behaviour therapy (regime/therapy) | L3 IAPT Therapy |  | Specialist Perinatal Mental Health Community Service | L4 MHP Specialist |
| Improving Access to Psychological Therapies high intensity therapy (regime/therapy) | L3 IAPT Therapy |  | Eating Disorders/Dietetics Service (Retired 1 April 2020) | L4 MHP Specialist |
| Eye movement desensitization and reprocessing therapy (regime/therapy) | L3 IAPT Therapy |  | Neurodevelopment Team | L4 MHP Specialist |
| Interpersonal psychotherapy (regime/therapy) | L3 IAPT Therapy |  | Crisis Resolution Team/Home Treatment Service | L4 MHP Crisis |
| Couple therapy for depression (regime/therapy) | L3 IAPT Therapy |  | Crisis Resolution Team | L4 MHP Crisis |
| Psychodynamic psychotherapy (regime/therapy) | L3 IAPT Therapy |  | Home Treatment Service | L4 MHP Crisis |
| Mindfulness-based therapy (regime/therapy) | L3 IAPT Therapy |  | Walk-in Crisis Assessment Unit Service | L4 MHP Crisis |
| Group psychotherapy (regime/therapy) | L3 IAPT Therapy |  | Psychiatric Decision Unit Service | L4 MHP Crisis |
| Guided self-help (regime/therapy) | L3 IAPT Therapy |  | Acute Day Service | L4 MHP Crisis |
| Employment support (regime/therapy) | L3 IAPT Therapy |  | Crisis House Service | L4 MHP Crisis |
| Memory Services/Clinic | L4 MHP General |  | Enhanced/Intensive Support Service | L4 MHP Crisis |
| Day Care Service | L4 MHP General |  | 24/7 Crisis Response Line | L4 MHP Crisis |
| Community Mental Health Team - Functional | L4 MHP General |  | Health Based Place of Safety Service | L4 MHP Crisis |
| Community Mental Health Team - Organic | L4 MHP General |  | Primary Care Mental Health Service | L4 MHP Triage |
| Community Rehabilitation Service | L4 MHP General |  | Single Point of Access Service | L4 MHP Triage |
| General Psychiatry Service | L4 MHP General |  | Mental Health inpatient | L5 AWP Inpatient |

*Note that inpatients within the secondary mental health care were all considered within Level 6 and AWP inpatient were consider in level 5. GP: general practice. IAPT: Improving Access to Physiological Therapy. MHP: Mental Health Provide. AWP: Avon and Wilshire Mental Health Partnership NHS trust

Figure S1: Summary of Weekly Queue size for services modelled post ‘lockdown’ (mean and 95% confidence bands) for the simulation scenarios A


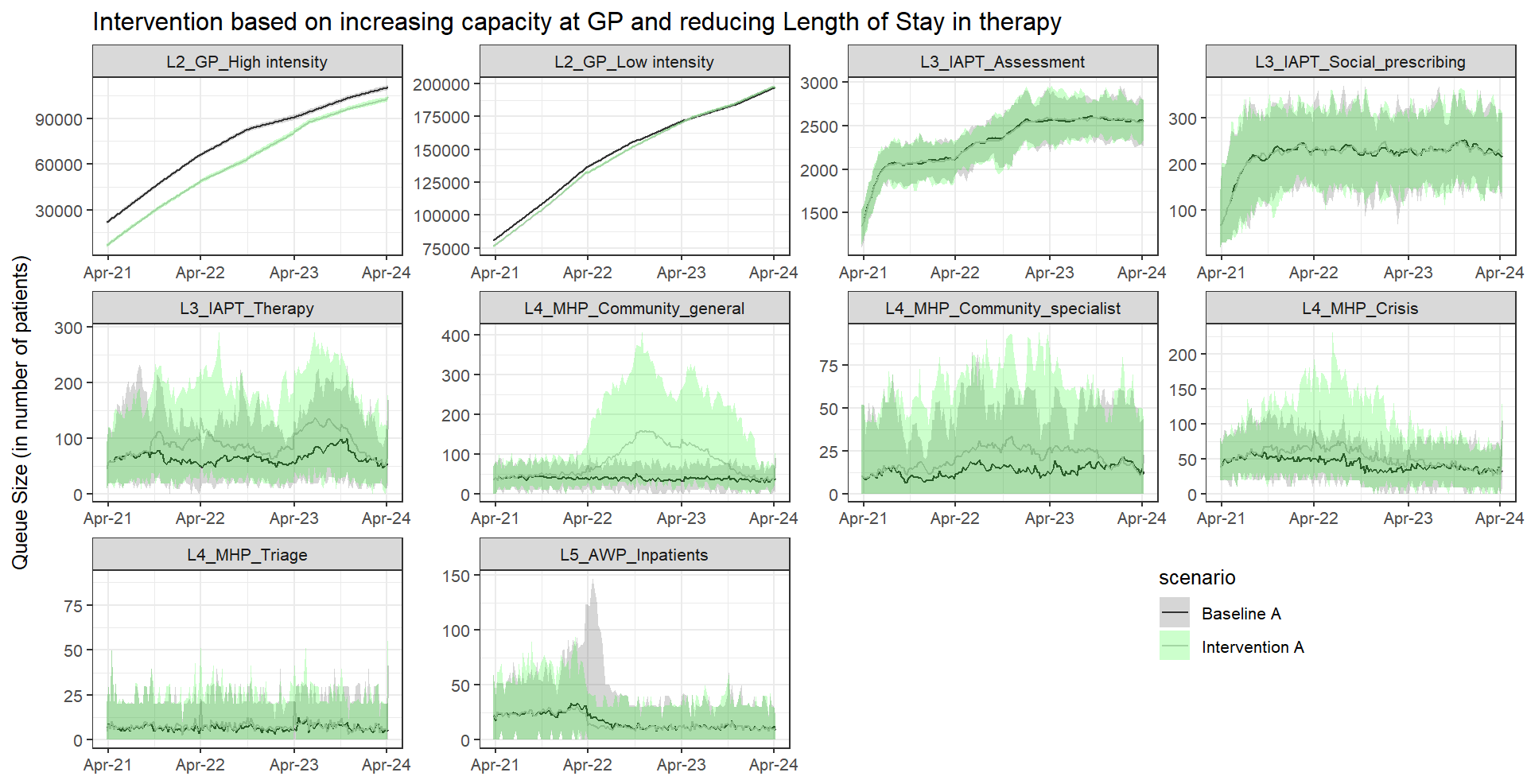


GP: general practice. IAPT: Improving Access to Physiological Therapy. MHP: Mental Health Provide. AWP: Avon and Wilshire Mental Health Partnership NHS trust

Figure S2: Summary of Weekly Queue size for services modelled post ‘lockdown’ (mean and 95% confidence bands) for the simulation scenarios B.


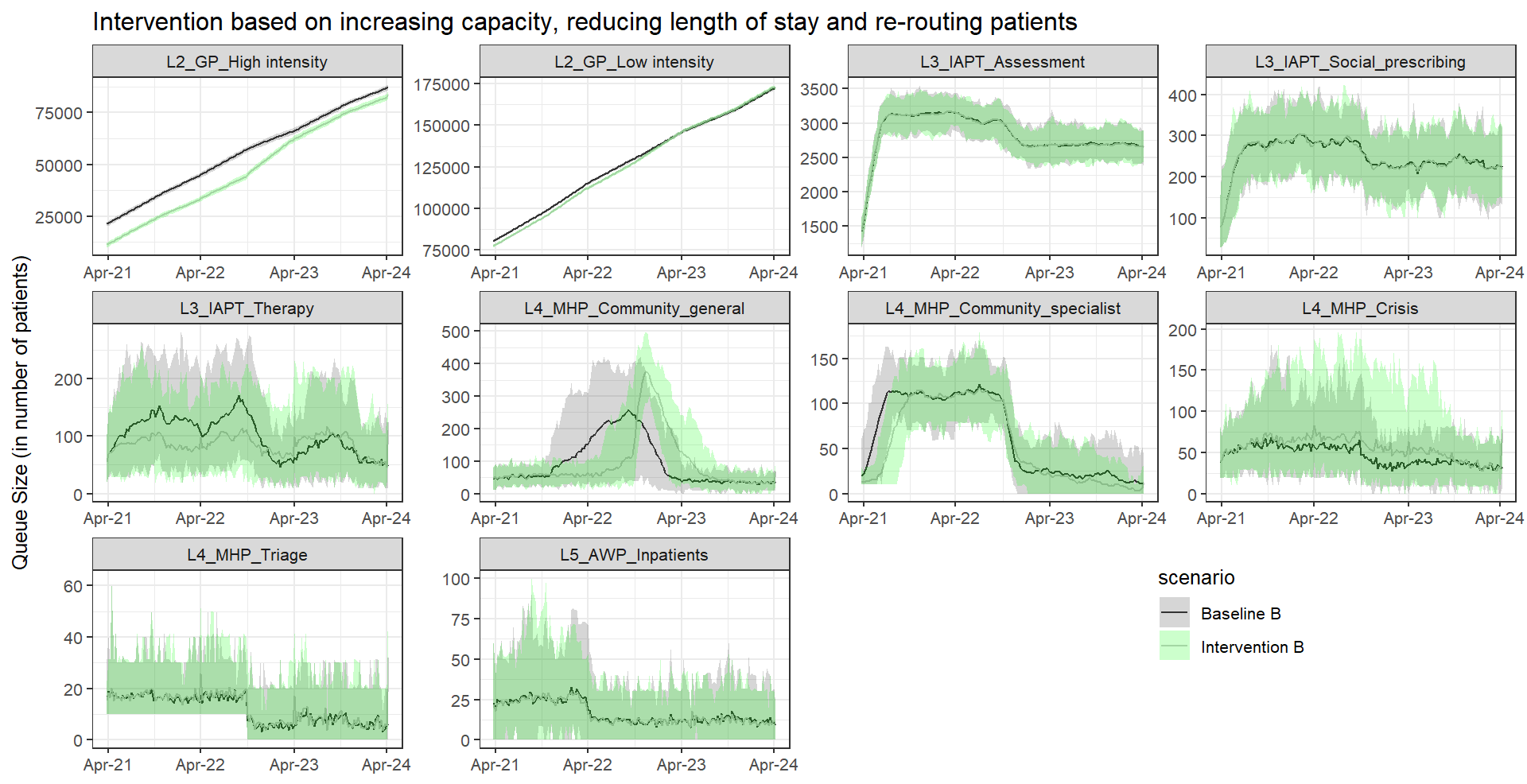


GP: general practice. IAPT: Improving Access to Physiological Therapy. MHP: Mental Health Provide. AWP: Avon and Wilshire Mental Health Partnership NHS trust
